# Maternal colonization with group B *Streptococcus* and antibiotic resistance in China: systematic review and meta-analyses

**DOI:** 10.1186/s12941-023-00553-7

**Published:** 2023-01-13

**Authors:** Jing Wang, Yan Zhang, Miao Lin, Junfeng Bao, Gaoying Wang, Ruirui Dong, Ping Zou, Yuejuan Chen, Na Li, Ting Zhang, Zhaoliang Su, Xiuzhen Pan

**Affiliations:** 1 Women’s Hospital of Jiangnan University, No. 48, Huaishu Lane, Wuxi, Jiangsu China; 2 Baoding No. 1 Hospital of TCM, Baoding, Hebei China; 3 College of Life Sciences, Nanjing Normal University, Nanjing, Jiangsu China; 4 Department of Microbiology, Hua Dong Research Institute for Medicine and Biotechnics, No. 293 Zhongshan East Road, Nanjing, Jiangsu China; 5 School of Medicine, Jiangsu University, No. 301 Xuefu Road, Zhenjiang, Jiangsu China

**Keywords:** Group B *streptococcus*, Pregnant, Colonization, Serotypes, Antibiotic resistance, China

## Abstract

**Background:**

Maternal rectovaginal colonization with group B *Streptococcus* (GBS) or *Streptococcus agalactiae* is the most common pathway for this disease during the perinatal period. This meta-analysis aimed to summarize existing data regarding maternal colonization, serotype profiles, and antibiotic resistance in China.

**Methods:**

Systematic literature reviews were conducted after searching 6 databases. Meta-analysis was applied to analyze colonization rate, serotype, and antimicrobial susceptibility of GBS clinical isolates in different regions of China. Summary estimates are presented using tables, funnel plots, forest plots, histograms, violin plots, and line plots.

**Results:**

The dataset regarding colonization included 52 articles and 195 303 pregnant women. Our estimate for maternal GBS colonization in China was 8.1% (95% confidence interval [CI] 7.2%–8.9%). Serotypes Ia, Ib, III, and V account for 95.9% of identified isolates. Serotype III, which is frequently associated with the hypervirulent clonal complex, accounts for 46.4%. Among the maternal GBS isolates using multilocus sequence typing (MLST), ST19 (25.7%, 289/1126) and ST10 (25.1%, 283/1126) were most common, followed by ST12 (12.4%, 140/1126), ST17 (4.8%, 54/1126), and ST651 (3.7%, 42/1126). GBS was highly resistant to tetracycline (75.1% [95% CI 74.0–76.3%]) and erythromycin (65.4% [95% CI 64.5–66.3%]) and generally susceptible to penicillin, ampicillin, vancomycin, ceftriaxone, and linezolid. Resistance rates of GBS to clindamycin and levofloxacin varied greatly (1.0–99.2% and 10.3–72.9%, respectively). A summary analysis of the bacterial drug resistance reports released by the China Antimicrobial Resistance Surveillance System (CARSS) in the past 5 years showed that the drug resistance rate of GBS to erythromycin, clindamycin, and levofloxacin decreased slowly from 2018 to 2020. However, the resistance rates of GBS to all 3 antibiotics increased slightly in 2021.

**Conclusions:**

The overall colonization rate in China was much lower than the global colonization rate (17.4%). Consistent with many original and review reports in other parts of the world, GBS was highly resistant to tetracycline. However, the resistance of GBS isolates in China to erythromycin and clindamycin was greater than in other countries. This paper provides important epidemiological information, to assist with prevention and treatment of GBS colonization in these women.

**Supplementary Information:**

The online version contains supplementary material available at 10.1186/s12941-023-00553-7.

## Background

**Fig. 1:**
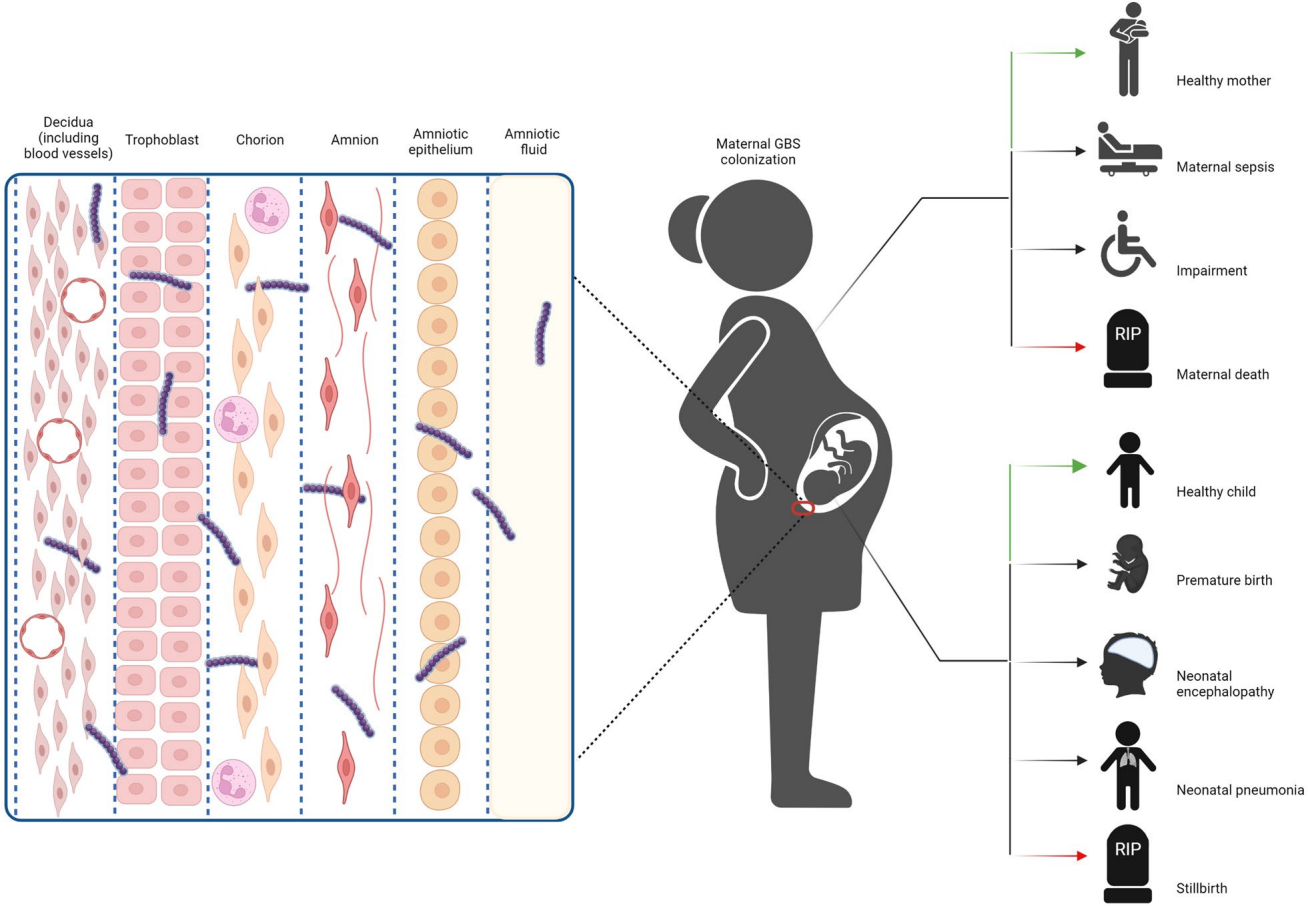
Maternal Group B *streptococcus* colonization in Group B *streptococcus* disease schema. Ascending Group B *streptococcus* infection during pregnancy ultimately leading to bacterial invasion of the amniotic cavity and fetus

Group B streptococcus (GBS) can colonize maternal gastrointestinal and reproductive tracts transiently, intermittently, or continuously [1, 2]. Under certain conditions, GBS can change from colonization to pathogenic. Because of its strong adsorption and penetration into the placental chorionic villi, vaginal GBS colonization during pregnancy is a risk for upstream infection and transmission to the newborn during delivery. Once GBS invades the amniotic cavity or comes into contact with the placenta, chorionic or placental inflammation may occur, which is usually associated with preterm birth and stillbirth [3–5]. In absence of prophylactic measures, GBS will be transmitted vertically to the infant through contaminated amniotic fluids and body fluid before or during delivery in approximately 50% of colonized women, causing invasive neonatal diseases such as pneumonia, neonatal sepsis, and neonatal meningitis [6–9]. Globally, > 500,000 newborns die from preterm birth each year, accounting for 44% of all deaths under the age of 5[10]. The majority of preterm births are caused by microbial infections, and of these, 10% are caused by GBS [11, 12]. GBS is a major causative agent of infectious diseases perinatally, and can cause neonatal death (Fig. 1).

Different rates of GBS colonization are observed worldwide, depending on geographic region and socioeconomic status [13]. For example, GBS colonization rate in pregnant women was 18.3% (95% CI 15.8%–20.7%) in the Americas, 15.4% (95% CI 12.2%–18.7%) in Southern Europe, 11.0% (95% CI 10.0%–12.0%) in Asia, and 18.2% (95% CI 16.1%–20.4%) in Africa [14]. In addition, the detection rate of GBS is also related to the sample timing, sampling site (vaginal sampling or rectovaginal sampling), and the microbiological methods. Therefore, in 2020, the American College of Obstetricians and Gynecologists (ACOG) updated the guidelines, suggesting that GBS screening should be performed by combined vaginal and rectal sampling to increase detection rates [15]. The sampled swabs should be cultured for 18–24 h in selective enrichment broth medium for enrichment and then inoculated into blood agar medium, followed by identification of GBS by techniques such as latex agglutination test, chromogenic culture, DNA probes or nucleic acid amplification tests. The ACOG recommended that the validity of GBS screening should be 5 weeks. If the GBS negative pregnant woman has not delivered for more than 5 weeks, the screening should be repeated [15]. To reduce the incidence of invasive GBS infection, the ACOG recommends vaginal-rectal GBS screening for all pregnant women at 35–37 weeks of gestation, and intrapartum antibiotic prophylaxis (IAP) for pregnant women with GBS colonization or risk factors [15]. This initiative significantly reduces the incidence of early-onset GBS sepsis by 50–80% [14]. However, the increased use of antibiotics has raised concerns about the emergence of drug resistance [8]. Therefore, understanding the prevalence, serotype distribution, and antimicrobial resistance of local GBS strains will help to formulate optimal prophylactic and treatment strategies.

China is a populous country with an annual birth population of more than 10 million. A low rate of GBS colonization in East Asia has been reported [14], nevertheless, the huge population base poses a substantial burden on infant invasive diseases caused by maternal colonization of GBS. Currently, data regarding GBS colonization, and the antimicrobial susceptibility of Chinese pregnant women exist only in the form of scattered literature reports. Therefore, this study conducted a systematic review and meta-analysis of GBS colonization, serotypes, serial types, antimicrobial susceptibility, and resistance trends in Chinese pregnant women, to assist with the development of optimal strategies for the prevention and treatment of GBS colonization in these women.

## Methods

### Study design

This systematic review and meta-analysis is reported according to the Preferred Reporting Items for Systematic reviews and Meta-Analyses 2020 (PRISMA 2020) statement published in 2021 [16]. We focused on the colonization and drug resistance of GBS in the reproductive tract of women in late pregnancy in China.

### Definitions

Group B *streptococcus* colonization refers to the isolation by culture of GBS from the vagina, rectum, or perianal area of a pregnant woman [14]. The resistance rate of antibiotics was based on the culture results, which is expressed as the number of resistant strains divided by the total number of tested strains. The results of phenotypic tests (E test, agar dilution) and genotype tests were accepted as the definition of drug resistance. According to the Clinical and Laboratory Standards Institute (CLSI) guidelines, the minimum inhibitory concentration (MIC) breakpoint for penicillin sensitive to GBS is ≤ 0.12 μg/mL, and the breakpoint of diameter of inhibition zone (DIZ) is ≥ 24 mm (10U). The MIC breakpoint for ampicillin sensitive to GBS is ≤ 0.25 μg/mL, and the breakpoint of DIZ is ≥ 24 mm (10 μg). The MIC breakpoint for ceftriaxone sensitive to GBS is ≤ 0.5 μg/mL, and the breakpoint of DIZ is ≥ 24 mm (30 μg).

China Antimicrobial Resistance Surveillance System (CARSS) was established by the National Health Commission of China in August 2005; it has a quality management center, three technical sub-centers, and monitoring centers in all provinces. Medical institutions regularly report experimental data via CARSS to achieve continuous monitoring of bacterial resistance changes of common pathogenic bacteria to antibacterial drugs. As of November 2021, CARSS membership had grown to 5570 medical institutions.

### Search strategy and selection criteria

Our search of published literature, dated 2001 up to 31st December 2020, included China National Knowledge Infrastructure (CNKI), Wanfang Data (WF), China Science and Technology Journal Database (CSTJ), Chinese Science Citation Database (CSCD), Chinese Social Sciences Citation Index (CSSCI), and China Biology Medicine disc (CBMdisc) using search terms relating to pregnancy/pregnant woman, group B *streptococcus*/*streptococcus agalactiae*/GBS, and/or drug sensitivity/antibiotic resistance. We screened titles and abstracts according to specified inclusion and exclusion criteria, followed by selection of full texts.

### Inclusion and exclusion criteria

Inclusion criteria: studies that described population and study design, reporting prevalence of GBS colonization in pregnant women (any gestation), as well as regarding antibiotic resistance or prevalence of the serotypes of colonizing isolates. Studies were included irrespective of sample source (vagina, rectum, perianal region, amniotic fluid, or midstream urine).

Exclusion criteria: we excluded studies involving nonpregnant women and newborn babies, where results for pregnant women could not be separately extracted. Moreover, comments, editorials, and reviews without estimating the proportion/prevalence and/or antibiotic resistance patterns and/or the serotype profiles among pregnant women colonized with GBS were excluded.

### Data abstraction

Researchers extracted data into excel spreadsheet form and checked the data. Disagreements were resolved by consensus. From each included study, the following parameters were extracted: publication year, study period, province and city, number of participants, GBS colonization, specimen type, serotype profiles of the isolates, and antibiotic resistance.

### Quality assessment

Quality evaluation was performed by two researchers independently, using an adapted version of the tool proposed by the Joanna Briggs Institute tool for critical appraisal of prevalence studies (Additional file 1: Table S1). After assessing the quality of each study, a composite quality score (0–10 points) was assigned. Studies scoring ≥ 5 were judged to be of moderate to high quality. A third reviewer adjudicated in any case of disagreement.

### Statistical analysis

We performed a meta-analysis to assess maternal GBS colonization rates and drug resistance from the national to the provincial level. The extracted data were exported to Stata v.14 (Statacorp LLC, Texas, USA) for analysis. The magnitude of heterogeneity between included studies was quantitatively measured by the heterogeneity index (I^2^ statistics) and the significance of heterogeneity by a P-value of I^2^; P ≤ 0.05 indicated statistical heterogeneity. The level of heterogeneity was defined as low (I^2^ = 25%), moderate (I^2^ = 50%), and high (I^2^ = 75%). A random-effects model was selected for I^2^ statistics > 50%, and a fixed-effects model was chosen for I^2^ statistics < 50%. Publication bias was visually evaluated using funnel plots, and then by Egger’s and Begg's statistics. A P-value ≤ 0.05 was considered indicative of the presence of statistically significant publication bias. Descriptive analysis was performed to investigate the distribution of serotypes. The results were presented in the form of tables, funnel plots, forest plots, histograms, violin plots, and line plots.

## Results

### Study characteristics

**Fig. 2:**
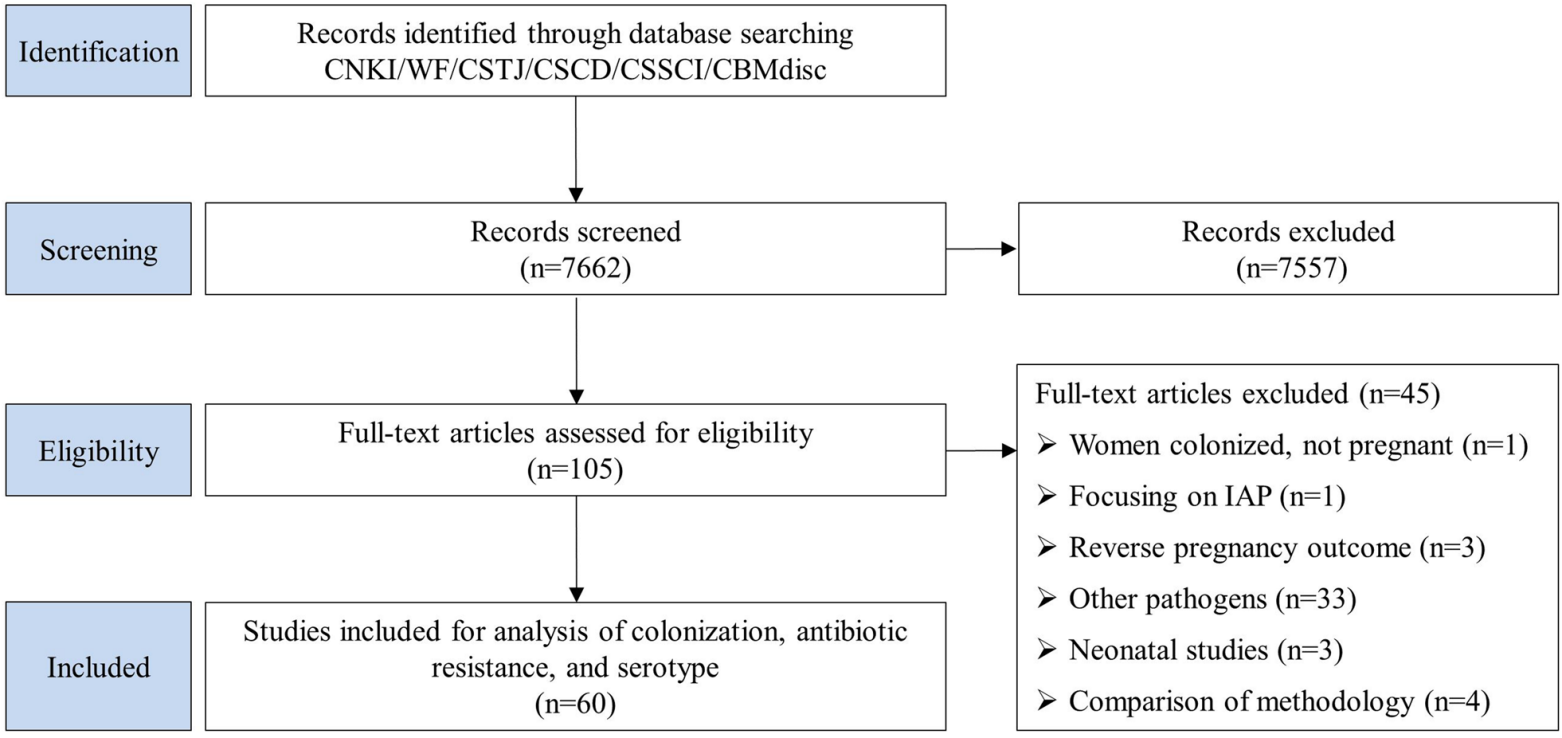
Data search and included studies for maternal Group B *streptococcus* colonization, antimicrobial resistance, and serotype. CNKI: China National Knowledge Infrastructure; WF: Wanfang Data; CSTJ: China Science and Technology Journal Database; CSCD: Chinese Science Citation Database; CSSCI: Chinese Social Sciences Citation Index; CBMdisc: China Biology Medicine disc

We identified 7662 articles, 105 of which were retained for review of full texts after title and abstract screening. The process of selection is detailed in Fig. 2. The final analysis included 60 studies, of which 52 reported maternal GBS colonization prevalence, 8 reported data on maternal colonizing serotypes, and 60 studies were included in sensitivity analyses (Additional file 2: Table S2, Additional file 3: Table S3).

### Quality assessment

There were no disagreements in the quality assessment of the included literature. Fifty-two (86.7%) studies were of high quality and eight (13.3%) studies were of medium quality. The number of samples included in the literature and the description of instruments and reagents, specimen collection, strain isolation and identification, and drug sensitivity tests were adequate.

### Risk of bias in included studies

**Fig. 3:**
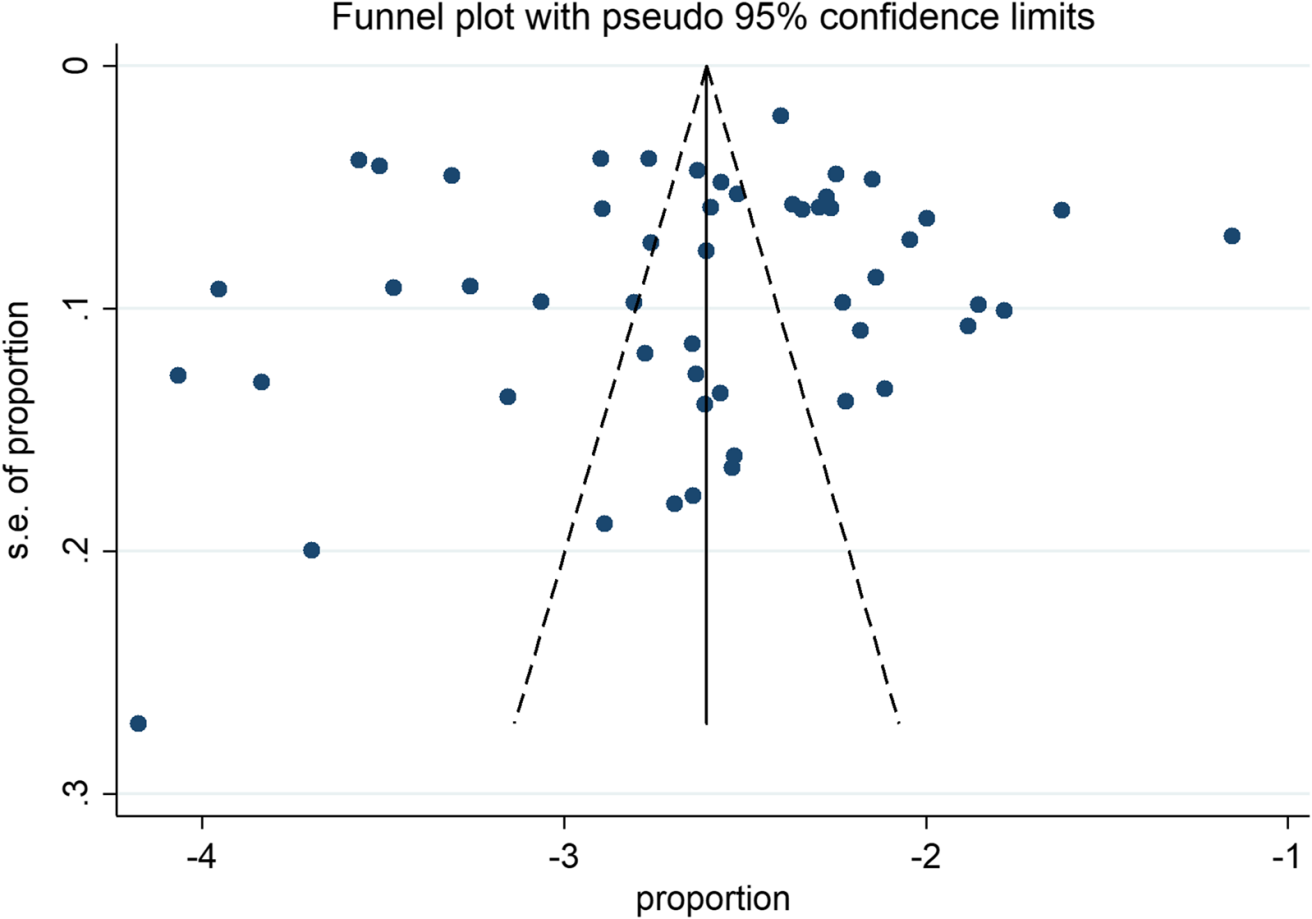
Funnel plot showing symmetrical distribution of the studies in China since 2010 to 2020. Funnel plots were drawn based on Group B *streptococcus* colonization rate. The results showed that the symmetry of the left–right distribution of each research site was good, and no visual evidence of publication bias was revealed

Funnel plots were drawn based on GBS colonization rate (Fig. 3). The results showed that the symmetry of the left–right distribution of each research site was good, and no visual evidence of publication bias was revealed. Egger's and Begg's test were used to detect publication bias, with P values of 0.855 and 0.240, respectively (Table 1), suggesting that there was no publication bias in this study.

**Table 1:** Begg's and Egger's tests for publication bias of maternal colonization with Group B *streptococcus* in China since 2010 to 2020

Begg's Test Adj. Kendall's Score (P-Q) = − 150 Std. Dev. of Score = 126.73 Number of Studies = 52 z = − 1.18 Pr >|z| = 0.237 z = 1.18 (continuity corrected) Pr > |z| = 0.240* (continuity corrected) Egger's test Number of Studies = 52	

Std_Eff	Coef	Std. Err	t	P >|t|	[95% Conf. Interval]	
Slope	− 2.581529	.1494765	− 17.27	0.000	− 2.881762	− 2.281297
Bias	− .4364169	2.37561	− 0.18	0.855*	− 5.20797	4.335135

*Begg's test and Egger's test are common statistical tests for quantitative evaluation of publication bias. P > 0.05 was considered as no publication bias. In this study, the results of Begg's test were P = 0.24 and Egger's test was P = 0.855, suggesting no significant publication bias

### Study characteristics

This review included data on colonization prevalence of 195,303 pregnant women with a total of 12,151 GBS isolates, including 11,460 items of drug sensitivity data and 1462 of serotyping data (1195 were typed by molecular methods and 267 by the latex agglutination method). Specimen collection was performed according to the recommended sampling methods: At 35–37 gestational weeks, vaginal secretions were sampled from the lower 1/3 of the vagina and rectal secretions were taken from the upper 2–3 cm of the anal sphincter. The specimens were placed in sterile tubes and immediately sent for examination.

**Fig. 4:**
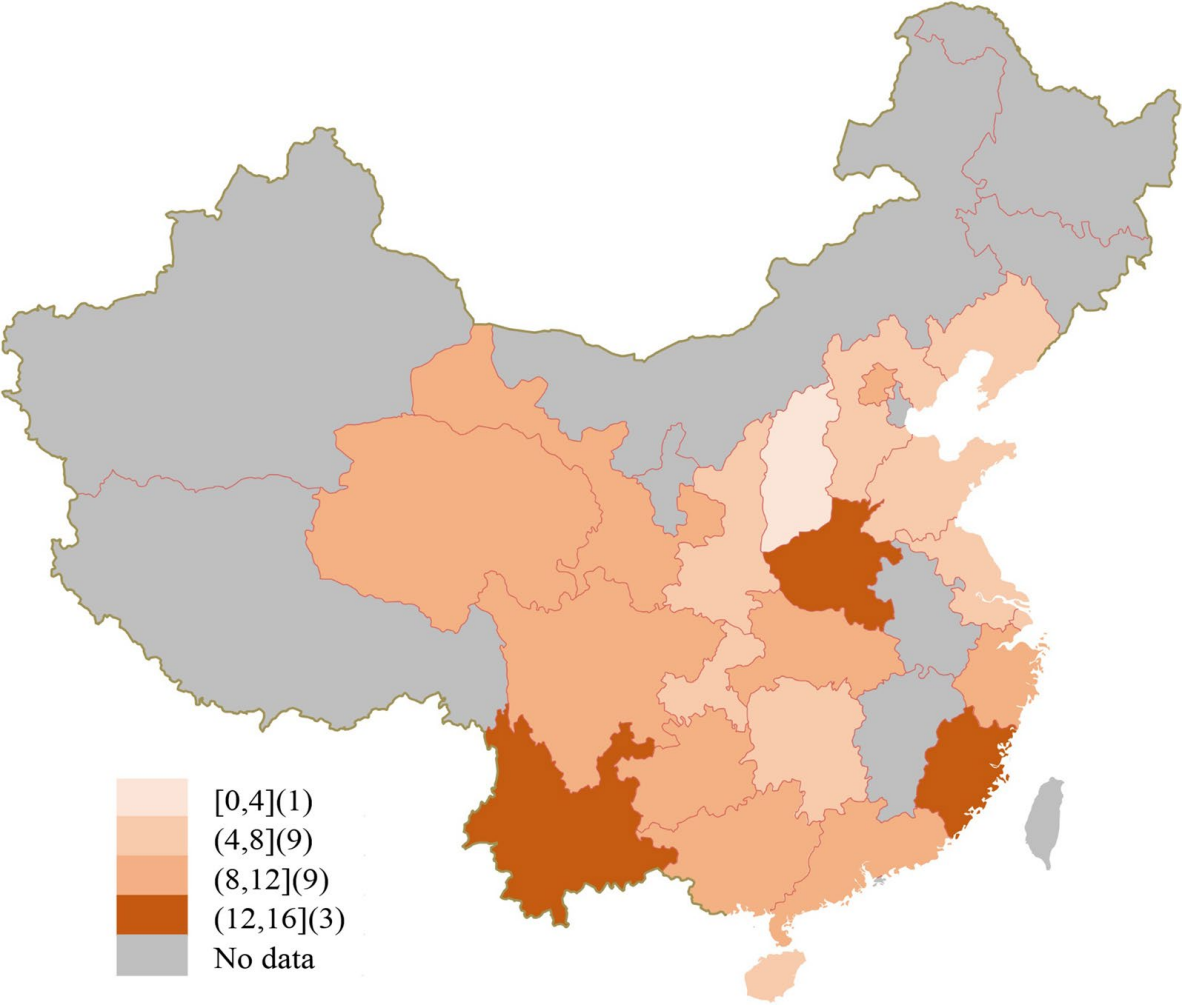
Geographic distribution of included data, showing the range of number of studies per province in China from 2010 to 2020. Borders of countries/territories on the map do not imply any political statement

Geographical distribution of colonization prevalence data was mostly concentrated in Beijing, Shanghai, Guangdong, Zhejiang, Hunan, Jiangsu, Shaanxi, and Sichuan. The developed regions (first-tier cities, new first-tier cities, and second-tier cities) reported the most. Other regions including Inner Mongolia Autonomous Region, Tibet Autonomous Region, Ningxia Hui Autonomous Region, Xinjiang Uygur Autonomous Region, Jilin Province, Heilongjiang Province, Anhui Province, Jiangxi Province, and other regions lacked relevant data (Fig. 4).

### Maternal GBS colonization prevalence: meta-analyses of reported data

**Fig. 5:**
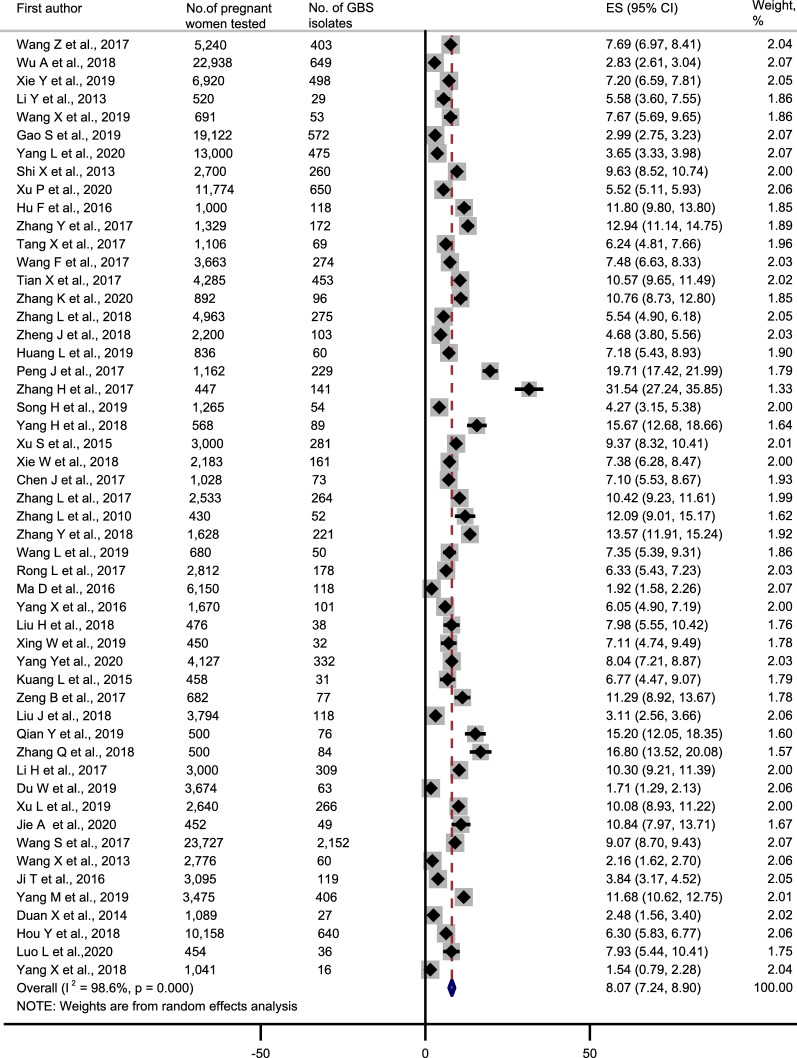
Forest plot showing the pooled estimates of the maternal Group B *streptococcus* colonization proportions in China since 2010 to 2020. The data of colonization were exported to Stata v.14. The red vertical dashed line represents an intuitive assessment of heterogeneity of the studies. If the vertical line can be drawn, forest plots indicate that all studies are sufficiently similar to be included in the meta-analysis. ES: effect size; 95% CI: 95% confidence interval; GBS: group B *Streptococcus*. Error bars indicate 95% CIs

Including all 52 studies, the overall prevalence of maternal GBS colonization in China was 8.1% (95% CI 7.2–8.9%) (Fig. 5). Prevalence was highest in Henan province (15.7% [95% CI 12.7–18.7%]) and lowest in Shanxi Province (1.7% [95% CI 1.3%–2.1%]). Hebei (7.7% [95% CI 7.0–8.4%]), Hunan (7.3% [95% CI 6.4–8.2%]), Hainan (7.1% [95% CI 4.7–9.5%]), Guangdong (8.1% [95% CI 5.0–11.3%]), Zhejiang (8.2% [95% CI 6.7–9.8%]), and Sichuan (8.6% [95% CI 6.5–10.7%]) provinces had similar prevalence, with a slightly higher prevalence in Beijing (11.1% [95% CI 6.2–15.9%]), Fujian (13.4% [95% CI 1.1–25.7%]), Yunnan (13.4% [95% CI 7.0–19.7%]), and seemingly lower prevalence in Shandong (4.3% [95% CI 3.2–5.4%]) and Shanxi (5.2% [95% CI 1.7–8.6%]) (Table 2).

**Table 2:** Maternal Group B *streptococcus* colonization prevalence results from meta-analyses with reported data in China from 2010 to 2020

Provinces/Regions (no. of studies)	Cities providing data (no. of studies)	No. of pregnant women tested	No. of GBS isolates	Reported prevalence, %	95% Confidence interval	I^2^ (%)^a^	p-value
Hebei (1)	Tangshan (1)	5240	403	7.7	7.0–8.4	–	–
Shanxi (3)	Xian (3)	30,378	1176	5.2	1.7–8.6	98.9	< 0.001
Liaoning (2)	Shenyang (2)	19,813	625	5.2	0.6–9.8	95.2	< 0.001
Jiangsu (2)	Nanjing (1) Suzhou (1)	15,700	735	6.6	0.8–12.5	99.0	< 0.001
Zhejiang (9)	Jiaxing (1) Jinhua (1) Lishui (1) Ningbo (1) Quzhou (1) Shaoxing (1) Wenzhou (3)	31,212	2210	8.2	6.7–9.8	96.4	< 0.001
Fujian (2)	Fuzhou (1) Quanzhou (1)	1998	289	13.4	1.1–25.7	98.6	< 0.001
Shandong (1)	Qingdao (1)	1265	54	4.3	3.2–5.4	–	–
Henan (1)	Pingdingshan (1)	568	89	15.7	12.7–18.7	–	–
Hubei (1)	Xianning (1)	3000	281	9.4	8.3–10.4	–	–
Hunan (2)	Changsha (1) Zhuzhou (1)	3211	234	7.3	6.4–8.2	0.0	0.779
Guangdong (8)	Dongguan (2) Foshan (1) Guangzhou (3) Huizhou (2)	16,379	1022	8.1	5.0–11.3	98.5	< 0.001
Hainan (1)	Haikou (1)	450	32	7.1	4.7–9.5	–	–
Sichuan (3)	Chengdu (2) Mianyang (1)	5267	440	8.6	6.5–10.7	75.4	0.017
Guizhou (2)	Guiyang (1) Xingyi (1)	4294	194	9.1	− 2.8 to 20.9	98.2	< 0.001
Yunnan (2)	Kunming (1) Qujing (1)	3500	393	13.4	7.0–19.7	92.7	< 0.001
Shanxi (1)	Hanzhong (1)	3674	63	1.7	1.3–2.1	–	–
Gansu (1)	Lanzhou (1)	2640	266	10.1	8.9–11.2	–	–
Qinghai (1)	Xining (1)	452	49	10.8	8.0–13.7	–	–
Guangxi (1)	Guangxi (1)	23,727	2151	9.1	8.7–9.4	–	–
Beijing (3)	Beijing (3)	6318	320	11.1	6.2–15.9	98.9	< 0.001
Shanghai (3)	Shanghai (3)	14,722	1073	6.8	2.6–11.0	98.8	< 0.001
Chongqing (2)	Chongqing (2)	1495	52	4.6	− 1.6 to 10.9	95.7	< 0.001
Overall	–	195,303	12,151	8.1	7.2–8.9	98.6	< 0.001

^a^Chi square

### Serotype distribution

**Fig. 6:**
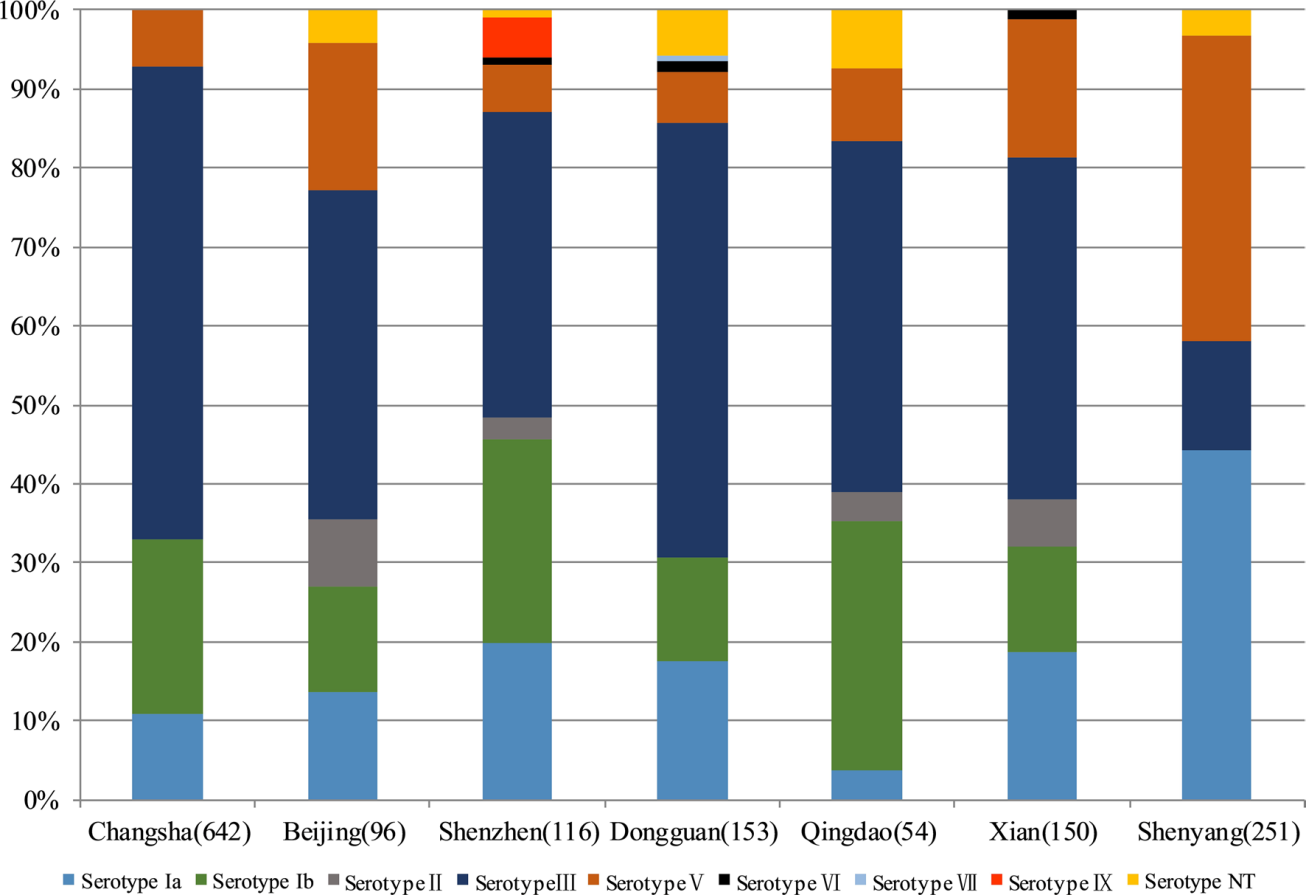
Maternal Group B *streptococcus* colonizing serotype distribution in China. Ia, Ib, III, and V predominated, accounting for 95.9% of the reported serotypes

Eight studies from 7 cities published the distribution of GBS serotypes. Ia, Ib, III, and V predominated, accounting for 95.9% of the reported serotypes. However, variation existed in the reported prevalence of serotype III. Compared to an overall serotype III prevalence of 46.4%, Changsha (60.0% of colonized women [95% CI 56.2–63.8%]) and Dongguan (54.9% [95% CI 46.9%–62.9%]), had a higher reported prevalence of serotype III. However, in Shenyang, the most common serotype was not III (13.9% [95% CI 9.6–18.3%]), but Ia (44.2% [95% CI 38.0–50.4%]) (Fig. 6).

### Multilocus sequence typing

Three articles reported MLST typing. Among the 1126 GBS isolates included in the former articles, 995 were clearly genotyped, and a total of 18 sequence types (STs) were reported. The major STs were ST19 (25.7%, 289/1126) and ST10 (25.1%, 283/1126), followed by ST12 (12.4%, 140/1126), ST17 (4.8%, 54/1126), ST651 (3.7%, 42/1126), and 13 other sequence types (16.6%, 187), and undetermined types (11.8%, 133).

### Drug resistance to antibiotics

**Fig. 7:**
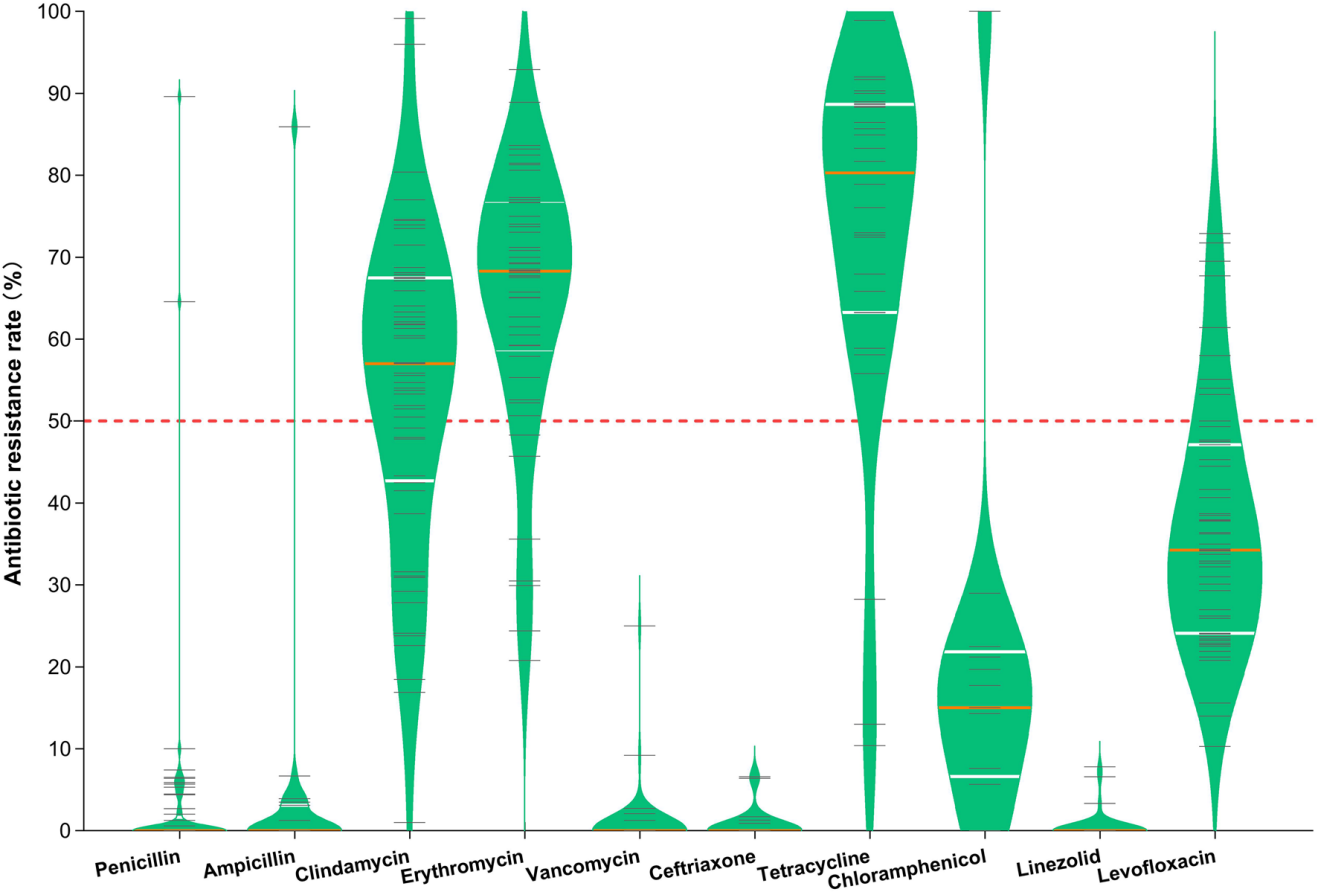
Violin plot showing the resistance of Group B *streptococcus* to antibiotics in China from 2008 to 2019. The short gray line represents the drug resistance data. The white and orange lines equate the data into 4 parts, where the orange line represents the median. Group B *streptococcus* was generally susceptible to penicillin, ampicillin, vancomycin, ceftriaxone, and linezolid, and was highly resistant to tetracycline and erythromycin

Sixty articles reported on the resistance of 11 460 GBS isolates. GBS was highly resistant to tetracycline (75.1% [95% CI 74.0–76.3%]) and erythromycin (65.4% [95% CI 64.5–66.3%]) (Fig. 7). Resistance rates of GBS to clindamycin and levofloxacin varied greatly (1.0–99.2% and 10.3–72.9%, respectively). GBS was generally susceptible to penicillin, ampicillin, vancomycin, ceftriaxone, and linezolid, and the antibiotic resistance rates were 1.6% [95% CI 1.3–1.8%], 0.7% [95% CI 0.5–0.9%], 0.3% [95% CI 0.2–0.4%], 0.7% [95% CI 0.5–0.9%], and 0.3% [95% CI 0.1–0.4%], respectively (Fig. 7).

This study further analyzed GBS resistance data from 7 administrative geographic regions in China. The greater variation in resistance was for clindamycin and levofloxacin, with the highest rate of resistance to clindamycin in the Northeast [74.3% (95% CI 69.4–79.3%)]. The lowest resistance rate to clindamycin was in northern China [33.6% (95% CI 30.7–36.4%)]. Regions with the highest and lowest resistance rates to levofloxacin were in Northeast China [67.7% (95% CI 61.9–73.6%)] and South China [24.0% (95% CI 21.7–26.3%)]. Resistance rates to erythromycin and tetracycline were high in all regions, with the highest rates of erythromycin resistance among GBS isolates in Northeast and North China, 82.2% (95% CI 77.9%–86.6%) and 71.5% (95% CI 68.8%–74.2%), respectively. In South China, GBS isolates were even more resistant to tetracycline [83.4% (95% CI 80.4%–86.4%)] (Table 3).

**Table 3:** Drug sensitivity analysis of Group B *Streptococcus* in different regions of China from 2008 to 2019

Administrative geographic division of China	Pooled prevalence of antibiotic resistance, % (95% confidence interval)									
	P	AMP	DA	E	VA	CTRX	TE	C	LZD	LEV
North China	2.7 (1.7, 3.8)	1.1 (0.2, 2.0)	33.6 (30.7. 36.4)	71.5 (68.8. 74.2)	0.2 (− 0.1, 0.5)	4.4 (1.6, 7.3)	77.5 (73.8, 81.3)	6.9 (4.6, 9.2)	0.0	46.2 (43.0, 49.4)
Northeast China	0.0	–	74.3 (69.4, 79.3)	82.2 (77.9. 86.6)	0.0	–	55.8 (49.6,62.0)	–	–	67.7 (61.9,73.6)
East China	2.6 (2.1, 3.0)	1.0 (0.7, 1.4)	51.4 (49.9, 52.9)	66.8 (65.4. 68.3)	0.4 (0.2, 0.6)	0.1 (− 0.6,1.5)	77.3 (75.5, 79.0)	50.9 (47.9,54.0)	0.3 (0.2, 0.5)	38.6 (37.2, 40.1)
Central China	0.4 (0, 0.7)	0.0	59.1 (56.3, 61.8)	54.1 (51.3. 56.9)	0.2 (− 0.1, 0.4)	0.0	65.5 (62.4,68.7)	2.2 (1.3, 3.2)	0.0	36.0 (33.4, 38.7)
South China	0.5 (0.1, 0.8)	0.0	55.8 (53.0, 58.5)	55.1 (51.9. 58.2)	0.0	0.0	83.4 (80.4, 86.4)	14.3 (4.8,23.7)	0.0	24.0 (21.7, 26.3)
Southwest China	0.5 (0.1, 0.8)	0.8 (− 0.1, 1.7)	61.5 (59.2, 63.7)	66.4 (64.2, 68.5)	0.5 (0.1, 0.8)	0.9 (− 0.9,2.8)	77.2 (75.0, 79.5)	16.2 (13.8, 18.6)	0.6 (0.1, 1.1)	37.2 (35.0, 39.5)
Northwest China	0.1 (− 0.1, 0.4)	0.0	65.1 (62.4, 67.8)	69.9 (67.3. 72.6)	0.0	0.0	–	–	0.0	47.9 (45.0, 50.7)

P: penicillin; AMP: ampicillin; DA: clindamycin; E: erythromycin; VA: vancomycin; CTRX: ceftriaxone; TE: Tetracycline; C: chloramphenicol; LZD: linezolid; LEV: levofloxacin

### Drug resistance trends reported by CARSS

**Fig. 8:**
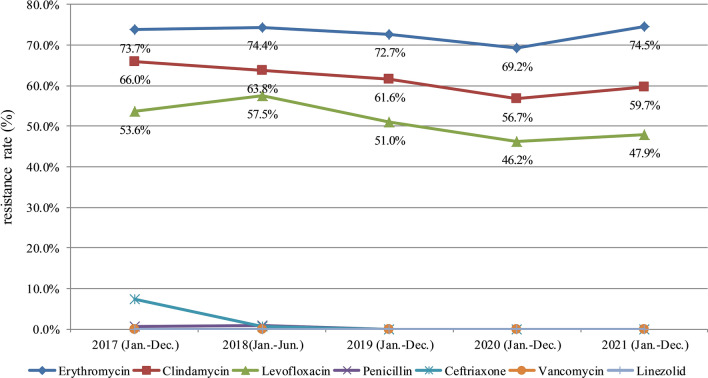
Antimicrobial resistance trends reported by China Antimicrobial Resistance Surveillance System (2017 to 2021). Resistance rates of Group B *streptococcus* to erythromycin, clindamycin, and levofloxacin all showed a slow decreasing trend from 2018 to 2020. However, Group B *streptococcus* resistance rates to all 3 antimicrobial drugs increased slightly in 2021

This paper summarizes GBS drug resistance in the monitoring report of CARSS over 5 recent years. Resistance rates of erythromycin, clindamycin and levofloxacin in different years were significantly different (P < 0.001). Resistance rates of GBS to erythromycin, clindamycin, and levofloxacin all showed a slow decreasing trend from 2018 to 2020. Statistical analysis was completed through pairwise comparison between various years. The results showed that the resistance rates of erythromycin, clindamycin and levofloxacin in 2020 were significantly lower than those in 2017, 2018 and 2019, respectively (P < 0.05). However, GBS resistance rates to erythromycin and clindamycin increased in 2021: resistance rates of both erythromycin and clindamycin in 2021 were higher than those in 2020 (P < 0.05). Although the rate of levofloxacin resistance increased in 2021 compared to 2020 (from 46.2% to 47.9%), the difference was not statistically significant, P > 0.05 (Table 4). The report showed that GBS was still susceptible to penicillin, ceftriaxone, vancomycin, and linezolid (Fig. 8).

**Table 4:** Comparison of drug resistance rates from 2017 to 2021 of China

Antibiotics		2017	2018	2019	2020	2021	χ^2^	p-value
Erythromycin	No. of GBS isolates	2218	1373	2774	3039	3764	39.333	< 0.001
	Drug resistance rate (%)	73.69	74.38	72.69	69.21^abc^	74.51^d^		
Clindamycin	No. of GBS isolates	1987	1178	2351	2409	3016	75.857	< 0.001
	Drug resistance rate (%)	66.01	63.81	61.61^a^	56.71^abc^	59.70^abd^		
Levofloxacin	No. of GBS isolates	1613	1061	1946	2029	2420	92.400	< 0.001
	Drug resistance rate (%)	53.59	57.48^a^	51.00^ab^	46.21^abc^	47.90^abc^		

^a^Compared with 2017, P < 0.05. ^b^Compared with 2018, P < 0.05. ^c^Compared with 2019, P < 0.05. ^d^Compared with 2020, P < 0.05

## Discussions

China has delivered 10.03 million to 17.86 million births each year in the past decade. Although the level of perinatal health care is constantly improving, the burden of neonatal invasive GBS disease in China remains high. Therefore, a systematic review of GBS colonization rates, serotype distribution, antimicrobial susceptibility, and resistance trend analysis of GBS in the recto-vagina of pregnant women in various regions of China can support the formulation of optimal strategies for the prevention and treatment of GBS colonization in pregnant women in China. To date, data above in China are small-scale and scattered. This paper is the first summary of data from 60 Chinese clinical studies, including colonization prevalence data from 195 303 Chinese pregnancies, 1462 serotyping data, and 11 460 drug susceptibility data, which can objectively reflect maternal GBS colonization and drug resistance.

In this study, the overall colonization rate of GBS among pregnant women in China was 8.1% (95% CI 7.2–8.9%), which was far lower than the global assessment of 17.4% (95% CI 16.3–18.5%) [14], and also lower than the colonization rate of GBS among pregnant women in Asia, 11.6% (95% CI 10.5–12.7%). A meta-analysis study analyzing a dataset including 85 countries and 299 924 pregnant women reported a GBS colonization rate of 11% (95% CI 10–13%) among Chinese women [14], which was higher than the overall colonization rate of 8.1% in this study. The reason for this difference may be because this meta-analysis included only a few Chinese reports from 2015 to 2017. Insufficient data may cause bias in overall GBS colonization rates. The present study contains GBS colonization data from 37 cities in China, which more objectively and truly reflects the overall colonization rate of GBS among pregnant women.

Capsular polysaccharide (CPS) is an important virulence factor of GBS. Based on CPS, GBS can be classified into 10 serotypes: Ia, Ib, and II–IX. The virulence and clinical manifestations of different serotypes are different [17]. This study reports data on the distribution of 8 serotypes in 7 Chinese cities, and all 10 serotypes of GBS were detected. Data analysis showed that Ia, Ib, III, and V were most common, accounting for 95.9% of all reported serotypes. This result is consistent with data evaluated globally: Ia, Ib, II, III, and V accounted for 98% of all serotypes [14]. The distribution of serotypes was associated with geographic location and ethnic variability. In Central America and Southeast Asia, the prevalence of serotype III associated with invasive disease was 11% (95% CI 7–14%) and 12% (95% CI 6–18%) respectively [14]. Compared to the overall prevalence of serotype III of 25% assessed globally [14], in China it was 46.4%. Higher serotype III prevalence rates of 60% (95% CI 56.2–63.8%) and 54.9% (95% CI 46.9–62.9%) were reported in some areas of China such as Changsha and Dongguan. Notably, there were significant regional differences in the definitive serotypes. In the Shenyang area, the most common serotype was not serotype III (13.9% [95% CI 9.6–18.3%), but Ia (44.2% [95% CI 38.0–50.4%). Similarly, the predominant serotype in southeastern Iran is III (41.8%) [18], while in northwestern Iran it is types V (19.5%) and Ia (17.6%) [19]. Because of the potential relevance of GBS serotypes to invasive disease and vaccine development, a comprehensive understanding of the prevalence of different serotypes across distinct regions in China is important for future vaccine implementation. It is worth noting that although the data in this study included all 10 serotypes of GBS, data on serotypes associated with maternal colonization in China are still limited.

Multilocus sequence typing (MLST) can amplify 7 housekeeping GBS genes using PCR, namely *adhP*, *pheS*, *atr*, *glnA*, *sdhA*, *glcK*, and *tkt*, to obtain the ST sequence types of different GBS isolates. MLST is a well-studied technical area, which describes a more diverse molecular profile than CPS and has its own prevalence pattern, providing a new perspective for understanding the prevalence characteristics of GBS at the genetic level. The main prevalent STs worldwide are ST-17 (20.4%), ST-1 (13.6%), ST-23 (12.6%), ST-19 (7.9%), and ST-10 (6.6%) [20]. This study included sequence typing data of 1126 isolates, among which ST19 (25.7%, 289/1126) and ST10 (25.1%, 283/1126) predominated. The prevalence of ST17 isolates closely associated with invasive infection was 4.8% in Sichuan, China. MLST typing can help clarify the epidemic strains of GBS and changes in drug resistance patterns. It has been suggested that III/ST19 isolates usually carry the *tetM* gene exhibiting high resistance to tetracycline, while Ib/ST10 isolates usually carry the *ermB* gene and exhibit high resistance to erythromycin [21], which may be related to the high resistance to tetracycline and erythromycin shown by Chinese GBS isolates. Although MLST can provide new perspectives on the epidemiological characteristics at the genetic level, there is currently insufficient data for MLST analysis of GBS, a problem that exists in China and world-wide.

Current interventions for perinatal GBS infection are limited to antibiotic therapy as IAP given at delivery (ACOG, 2020). ACOG recommends penicillin as the first-line antibiotic for the treatment of invasive GBS infection and IAP. With the widespread use of IAP for the prevention of early-onset GBS disease, the problem of GBS resistance to multiple antimicrobial drugs recommended by the Performance Standards for Antimicrobial Susceptibility Testing M100 has gradually arisen. Here, data from 60 manuscripts were pooled to analyze antibiotic resistance of 11,460 GBS isolates in China. Results indicated that Chinese pregnancy GBS isolates were sensitive to penicillin with a resistance rate of 1.6% (95% CI 1.3–1.8%), and penicillin is the preferred antibiotic regimen for prophylaxis of GBS infection. Despite this sensitivity, vigilance is required. A study from Japan reports that GBS penicillin resistance in Japan increased sharply from 2.3% to 14.7% between 2005 and 2013 [22]. Reduced sensitivity of GBS to penicillin has also been reported in Canada [23], Korea [24], and Germany [25]. Accordingly, it would be meaningful for GBS resistance monitoring if microbiology laboratories, while reporting the MIC of penicillin, ampicillin and ceftriaxone, could also focus on the data of MIC90 and MIC50 of the above drugs.

The Clinical and Laboratory Standards Institute recommends cefazolin for pregnant women with low-risk of allergic reactions to penicillin, and clindamycin for those with a high-risk of allergic reactions to penicillin. Clindamycin resistance shows wide variations globally. A report from Thailand recorded a 22.0% resistance rate to clindamycin in GBS [26]. Clindamycin resistance in the USA increased from 37.0% in 2011 to 43.2% in 2016 [27]. Data from this study showed that the highest resistance rate was observed in Northeast China [74.3% (95% CI 69.4–79.3%)], followed by Northwestern [65.1% (95% CI 62.4–67.8%)] and Southwestern China [61.5% (95% CI 59.2–63.7%)]. The lowest GBS resistance rate was observed in North China [33.6% (95% CI 30.7–36.4%)].

Although tetracycline is not commonly used to treat GBS infection, it has the highest resistance rate. Data from Africa showed a high resistance rate of 82.6% (95% CI 75.9–89.4%) to tetracycline in GBS isolates in pregnant African women [28]. The results of this study showed that GBS was highly resistant to tetracycline at 75.1% (95% CI 74.0–76.3%). In addition to tetracycline, Chinese GBS isolates were also highly resistant to erythromycin [65.4% (95% CI 64.5%–66.3%)]. Similar to the situation in China, a Korean report gave a resistance rate of 42.9–51.8% for GBS to erythromycin. A systematic evaluation based on global studies showed a 25% resistance rate to erythromycin in GBS [29]. Although erythromycin resistance rates are not as high in other regions; for example, 8.0% in Canada [30], 15% in England and Wales neonatal GBS isolates [31], and 20.8% in African pregnant women with GBS isolates [28], the ACOG concluded that erythromycin resistance rates continue to increase and that erythromycin has difficulty achieving effective drug concentrations in the amniotic fluid or fetus. Therefore, ACOG no longer recommends erythromycin and clindamycin as alternatives to penicillin in the new guidelines in 2020, but instead recommends cefazolin (low-risk of severe allergic reactions), clindamycin, or vancomycin (high-risk of severe allergic reactions). However, of concern is how to use vancomycin safely and rationally. If the vancomycin therapeutic dose is insufficient, it may cause bacterial selective pressure and lead to the emergence of vancomycin-mediated or resistant strains. Conversely, if the therapeutic dose is too high, there is a risk of nephrotoxicity. In addition, the macrolide-lincosamide-streptogramine (MLS) resistance phenotype should also be taken seriously by clinicians. MLS resistance can be divided into inducible (iMLS) resistance and constitutive (cMLS) resistance. In order to avoid irrational use of clindamycin by clinicians, the microbiology laboratory must use the D-test method to detect iMLS when the isolates are resistant to erythromycin. If D-test is positive, it shall be reported that the isolates are resistant to erythromycin and clindamycin. Otherwise, treatment with clindamycin may lead to failure.

China Antimicrobial Resistance Surveillance System was established in 2005, and its members cover more than 5570 medical institutions in China. Each medical institution regularly reports routine microbial drug sensitivity test data to the competent authorities via the bacterial drug resistance surveillance information system on a quarterly basis. The sensitivity and resistance rates of common clinical pathogenic bacteria to various types of antimicrobial drugs are calculated by CARSS every year to generate an annual bacterial drug resistance surveillance report. These reports can truly reflect the bacterial drug resistance levels in China. Here, we summarized GBS resistance data from the report of 2017–2021 to understand the resistance trend of GBS isolates in China. The results showed that Chinese GBS isolates demonstrate good susceptibility to penicillin, ceftriaxone, vancomycin, and linezolid from 2018 to 2020. The resistance rates of erythromycin, clindamycin, and levofloxacin all showed a slow decreasing trend, which may be related to the effectiveness achieved by medical institutions in recent years in actively implementing the national policy on the rational clinical application of antibacterial drugs and strengthening hospital infection control. However, in 2021, GBS showed a slight increase in the rate of resistance to erythromycin, clindamycin, and levofloxacin, which should arouse vigilance. We should list microbial resistance as an important issue, further strengthen the rational clinical application of antimicrobial drugs in hospitals, standardize the empirical use of drugs, and reduce the unindicated use of antimicrobial drugs, so as to delay the emergence of microbial resistance.

## Conclusions

The data generated from this systematic review and meta-analysis provide important epidemiological information. GBS colonization rate among pregnant women in China is much lower than that in pregnant women assessed globally, but the burden of neonatal invasive GBS disease remains high because of the large population base in China. Penicillin is still the preferred antibiotic regimen for prophylaxis of GBS infection. Of public concern is that although IAP has reduced the incidence of early-onset GBS sepsis by 50%–80%, the incidence of drug-resistant or non-GBS strains early-onset sepsis in preterm infants owing to IAP is increasing [32]. Therefore, we should list microbial drug resistance as an important issue, standardize empirical use of drugs, so as to delay the emergence of microbial resistance. Although serotype and MLST can provide new perspectives on the characteristics of GBS, there is currently insufficient data in China. Thus, more data on local GBS strains will help future vaccine implementation and formulate optimal prophylactic and treatment strategies.

## Supplementary Information

**Additional file 1: Table S1.** Quality assessment of included studies.

**Additional file 2: Table S2.** Characteristics of included studies.

**Additional file 3: Table S3.** References used in meta-analysis.

## Data Availability

All data analysed during this study are included in this published article and its supplementary table files.

## Abbreviations

GBS: Group B *Streptococcus*
MLST: Multilocus sequence typing
CARSS: China Antimicrobial Resistance Surveillance System
ACOG: American College of Obstetricians and Gynecologists
IAP: Intrapartum antibiotic prophylaxis
MIC: Minimum inhibitory concentration
DIZ: Diameter of inhibition zone
CNKI: China National Knowledge Infrastructure
WF: Wanfang Data
CSTJ: China Science and Technology Journal Database
CSCD: Chinese Science Citation Database
CSSCI: Chinese Social Sciences Citation Index
CBMdisc: China Biology Medicine disc
CPS: Capsular polysaccharide
CLSI: Clinical and Laboratory Standards Institute
