# Correction: Maternal colonization with group B *Streptococcus* and antibiotic resistance in China: systematic review and meta-analyses

**DOI:** 10.1186/s12941-023-00563-5

**Published:** 2023-02-22

**Authors:** Jing Wang, Yan Zhang, Miao Lin, Junfeng Bao, Gaoying Wang, Ruirui Dong, Ping Zou, Yuejuan Chen, Na Li, Ting Zhang, Zhaoliang Su, Xiuzhen Pan

**Affiliations:** 1 Women’s Hospital of Jiangnan University, No. 48, Huaishu Lane, Wuxi, Jiangsu China; 2 Baoding No. 1 Hospital of TCM, Baoding, Hebei China; 3 College of Life Sciences, Nanjing Normal University, Nanjing, Jiangsu China; 4 Department of Microbiology, Hua Dong Research Institute for Medicine and Biotechnics, No. 293 Zhongshan East Road, Nanjing, Jiangsu China; 5 School of Medicine, Jiangsu University, No. 301 Xuefu Road, Zhenjiang, Jiangsu China

**Correction: Ann Clin Microbiol Antimicrob (2023) 22:5** **https://doi.org/10.1186/s12941-023-00553-7**

Following publication of the original article [1], the authors noticed an error in the grant number under Funding section. This error does not affect any of the conclusions reported in the article. The updated funding section is given in this correction.

**Funding**

This work was supported by the National Natural Science Foundation of China (Grant Nos. 81701635, 82171674, 82072256), the Natural Science Foundation of Jiangsu Province (Grant No. BK20201129), the Wuxi City Health Committee top-notch talent (Grant No. BJ2020077).

The original article has been corrected.
