# Supplementary material for: Maternal colonization with group B Streptococcus and antibiotic resistance in China: systematic review and meta-analyses
Source: Ann Clin Microbiol Antimicrob. 2023 Jan 13;22:5. doi: 10.1186/s12941-023-00553-7 (PMC9837753; doi:10.1186/s12941-023-00553-7)
Supplement: Supplementary file 1 — Additional file 1: Table S1. Quality assessment of included studies. [file 12941_2023_553_MOESM1_ESM.docx]

**Table S1.** Quality Assessment of Included Studies

| Criteria* | Yes | No | Unclear | Not applicable |
| --- | --- | --- | --- | --- |
| 1. Was the sample representative of the target population? |  |  |  |  |
| 2. Were study participants recruited in an appropriate way? |  |  |  |  |
| 3. Was the samples size adequate? |  |  |  |  |
| 4. Were the study subjects and the setting described in detail? |  |  |  |  |
| 5. Was the data analysis conducted with sufficient coverage of the identified sample? |  |  |  |  |
| 6. Were objective, standard criteria used for the measurement of the condition? |  |  |  |  |
| 7. Was the condition measured reliably? |  |  |  |  |
| 8. Was there appropriate statistical analysis? |  |  |  |  |
| 9. Are all important confounding factors/subgroups/differences identified and accounted for? |  |  |  |  |
| 10. Were subpopulations identified using objective criteria? |  |  |  |  |

* The Joanna Briggs Institute Prevalence Critical Appraisal Tool. The 10 criteria used to assess the methodological quality of studies. These questions can be answered either with a yes, no, unclear, or not applicable.
