# Supplementary material for: Maternal colonization with group B Streptococcus and antibiotic resistance in China: systematic review and meta-analyses
Source: Ann Clin Microbiol Antimicrob. 2023 Jan 13;22:5. doi: 10.1186/s12941-023-00553-7 (PMC9837753; doi:10.1186/s12941-023-00553-7)
Supplement: Supplementary file 2 — Additional file 2: Table S2. Characteristics of included studies. [file 12941_2023_553_MOESM2_ESM.docx]

**Table S2.** Characteristics of included studies

| Reference | Region of China | Year of  publication | Year of data collection | Colonization | Serotype | MSLT^a^ | AMR^b^ | Study design |  |
| --- | --- | --- | --- | --- | --- | --- | --- | --- | --- |
|  |  |  |  |  |  |  |  |  |  |
| Wang Z et al. (1) | Tangshan | 2017 | 2013-2014 | Y | N | N | Y | R |  |
| Wu A et al. (2) | Xian | 2018 | 2013-2017 | Y | N | N | Y | R |  |
| Xie Y et al. (3) | Xian | 2019 | 2015-2017 | Y | Y | N | Y | R |  |
| Li Y et al. (4) | Xian | 2013 | 2011-2012 | Y | N | N | Y | R |  |
| Wang X et al. (5) | Shenyang | 2019 | 2017 | Y | N | N | Y | R |  |
| Gao S et al. (6) | Shenyang | 2019 | 2016-2017 | Y | Y | N | Y | R |  |
| Yang L et al. (7) | Nanjing | 2020 | 2016-2018 | Y | N | N | Y | R |  |
| Shi X et al. (8) | Suzhou | 2013 | 2010-2012 | Y | N | N | Y | R |  |
| Xu P et al. (9) | Jiaxing | 2020 | 2016-2017 | Y | N | N | Y | R |  |
| Hu F et al. (10) | Jinhua | 2016 | 2014-2016 | Y | N | N | Y | R |  |
| Zhang Y et al. (11) | Lishui | 2017 | 2014-2016 | Y | N | N | Y | R |  |
| Tang X et al. (12) | Ningbo | 2017 | 2013-2016 | Y | N | N | Y | R |  |
| Wang F et al. (13) | Quzhou | 2017 | 2014-2016 | Y | N | N | Y | R |  |
| Tian X et al. (14) | Shaoxing | 2017 | 2016 | Y | N | N | Y | R |  |
| Zhang K et al. (15) | Wenzhou | 2020 | 2017-2019 | Y | N | N | Y | R |  |
| Zhang L et al. (16) | Wenzhou | 2018 | 2016-2017 | Y | N | N | Y | R |  |
| Zheng J et al. (17) | Wenzhou | 2018 | 2016-2017 | Y | N | N | Y | R |  |
| Huang L et al. (18) | Fuzhou | 2019 | 2019 | Y | N | N | Y | R |  |
| Peng J et al. (19) | Quanzhou | 2017 | 2012-2016 | Y | N | N | Y | R |  |
| Zhang H et al. (20) | Beijing | 2017 | 2014-2016 | Y | N | N | Y | R |  |
| Song H et al. (21) | Qingdao | 2019 | 2017-2018 | Y | Y | N | Y | R |  |
| Yang H et al. (22) | Pingdingshan | 2018 | 2015-2017 | Y | N | N | Y | R |  |
| Xu S et al. (23) | Xianning | 2015 | 2009-2013 | Y | N | N | Y | R |  |
| Xie W et al. (24) | Changsha | 2018 | 2016-2017 | Y | N | N | Y | R |  |
| Chen J et al. (25) | Zhuzhou | 2017 | 2015 | Y | N | N | Y | R |  |
| Zhang L et al. (26) | Dongguan | 2017 | 2013-2014 | Y | Y | N | Y | R |  |
| Zhang L et al. (27) | Dongguan | 2010 | U | Y | N | N | Y | R |  |
| Zhang Y et al. (28) | Foshan | 2018 | 2017 | Y | N | N | Y | R |  |
| Wang L et al. (29) | Guangzhou | 2019 | 2018 | Y | N | N | Y | R |  |
| Rong L et al. (30) | Guangzhou | 2017 | 2015 | Y | N | N | Y | R |  |
| Ma D et al. (31) | Guangzhou | 2016 | 2013-2014 | Y | N | N | Y | R |  |
| Yang X et al. (32) | Huizhou | 2016 | 2014-2015 | Y | N | N | Y | R |  |
| Liu H et al. (33) | Huizhou | 2018 | 2014-2017 | Y | N | N | Y | R |  |
| Xing W et al. (34) | Haikou | 2019 | 2016-2018 | Y | N | N | Y | R |  |
| Yang Yet al. (35) | Chengdu | 2020 | 2018-2019 | Y | N | Y | Y | R |  |
| Kuang L et al. (36) | Chengdu | 2015 | 2012-2013 | Y | N | N | Y | R |  |
| Zeng B et al. (37) | Mianyang | 2017 | 2016-2017 | Y | N | N | Y | R |  |
| Liu J et al. (38) | Guiyang | 2018 | 2016-2017 | Y | N | N | Y | R |  |
| Qian Y et al. (39) | Xingyi | 2019 | 2016-2018 | Y | N | N | Y | R |  |
| Zhang Q et al. (40) | Kunming | 2018 | 2016-2017 | Y | N | N | Y | R |  |
| Li H et al. (41) | Qujing | 2017 | 2014-2016 | Y | N | N | Y | R |  |
| Du W et al. (42) | Hanzhong | 2019 | 2016-2017 | Y | N | N | Y | R |  |
| Xu L et al. (43) | Lanzhou | 2019 | 2017-2018 | Y | N | N | Y | R |  |
| Jie A et al. (44) | Xining | 2020 | 2017-2019 | Y | N | N | Y | R |  |
| Wang S et al. (45) | Nanning | 2017 | 2015-2016 | Y | N | N | Y | R |  |
| Wang X et al. (46) | Beijing | 2013 | 2011-2012 | Y | N | N | Y | R |  |
| Ji T et al. (47) | Beijing | 2016 | 2014-2015 | Y | N | N | Y | R |  |
| Yang M et al. (48) | Shanghai | 2019 | 2016-2017 | Y | N | Y | Y | R |  |
| Duan X et al. (49) | Shanghai | 2014 | 2014 | Y | N | N | Y | R |  |
| Hou Y et al. (50) | Shanghai | 2018 | 2016 | Y | N | N | Y | R |  |
| Luo L et al. (51) | Chongqing | 2020 | 2015-2018 | Y | N | N | Y | R |  |
| Yang X et al. (52) | Chongqing | 2018 | 2016 | Y | N | N | Y | R |  |
| Tan H et al. (53) | Changsha | 2020 | 2018-2019 | N | Y | Y | Y | R |  |
| Li D et al. (54) | Beijing | 2018 | 2016-2017 | N | Y | N | Y | R |  |
| Nie Set al. (55) | Shenzhen | 2018 | 2015-2016 | N | Y | N | Y | R |  |
| Liu J et al. (56) | Beijing | 2013 | 2009-2013 | N | N | N | Y | R |  |
| Su J et al. (57) | Shenzhen | 2016 | 2008-2015 | N | Y | N | Y | R |  |
| Wang F et al. (58) | Huizhou | 2016 | 2014-2016 | N | N | N | Y | R |  |
| Wu H et al. (59) | Dongying | 2016 | 2013-2015 | Y | N | N | Y | R |  |
| Liu J et al. (60) | Beijing | 2015 | 2011-2013 | N | N | N | Y | R |  |

^a^AMR, antimicrobial drug resistance; ^b^MLST, multilocus sequence typing; Y, yes; N, no; R, retrospective study; U, unknown (information not available).
