# Supplementary material for: Maternal colonization with group B Streptococcus and antibiotic resistance in China: systematic review and meta-analyses
Source: Ann Clin Microbiol Antimicrob. 2023 Jan 13;22:5. doi: 10.1186/s12941-023-00553-7 (PMC9837753; doi:10.1186/s12941-023-00553-7)
Supplement: Supplementary file 3 — Additional file 3: Table S3. References used in meta-analysis. [file 12941_2023_553_MOESM3_ESM.docx]

**Table S3.** References used in meta-analysis.

| No. | References |
| --- | --- |
|  |  |
| 1 | XIE Anxia, et al. Screening of group B Streptococcus in 452 women in late pregnaney . pregnaney outcomes , and analysis ofhigh-risk factors for colonization. Journal of Pathogen Biology,2020,15(08):967-970. |
| 2 | YANG Lindong, et al. Relationship of group B streptococcus colonization in late pregnancy with perinatal outcomes. Journal of Zhejiang University( Medical Sciences),2020,49(03):389-396. |
| 3 | TAN Hao, et al. Drug resistance of group B Streptococcus. Chin J Nosocomiol, 2020,30(16):2524-2527. |
| 4 | LUO Li, et al. Effect of Group B Streptococcus colonization on pregnancy outcome in 454 pregnant women. Journal of Chongqing Medical University, 2020,45(12):1805-1808. |
| 5 | XING Wei, et al. Relationship between group B streptococcal infection in perinatal period and pregnancy outcome. Chin J Nosocomiol, 2019,29(11):1704-1707. |
| 6 | LI Dong, et al. Antibiotic resistance and serotype distribution of group B Streptococci in pregnant women in Beijing. Journal of Capital Medical University, 2018,39(04):591-595. |
| 7 | WANG Xiaona, et al. The clinical effect of antibiotic treatment during pregnancy and maternity of pregnant women with B group streptococcal infection. Chin J Microecol, 2019,31(08):907-909-914. |
| 8 | YANG Mengnan, et al. Status of group B streptococcal infection during pregnancy and its effect on the maternal-neonatal outcomes. Prog Obstet Gynecol, 2019,28(03):173-177. |
| 9 | NIE Shuping, et al. Epidemiological characteristics of group B streptococcus colonized in pregnant women in Shenzhen City. Chin J Infect Control, 2018,17(06):522-526. |
| 10 | PENG Jie, et al. Infection situation and drug resistance of group B Streptococcus in urogenital tract of pregnant women. Chin J Nosocomiol, 2017,27(08):1841-1844. |
| 11 | YANG Yinyan, et al. Genotypes and drug resistance of group B Streptococcus causing infection in late pregnant women. Chin J Nosocomiol, 2020,30(16):2515-2519. |
| 12 | LI Hongying, et al. Study on the influence of group B streptococcal infection in pregnant women on the prognosis of mothers and infants. Chinese Journal of Practical Gynecology and Obstetrics, 2017,33(08):850-852. |
| 13 | ZHANG Lihua, et al. Serotypes and antimicrobial resistance profile of the group B Streptococcus isolates in perinatal pregnant women during the period from 2013 to 2014. Chin J Infect Chemother,2017,17(05):527-531. |
| 14 | YANG Xiaolan, et al. Clinical effect of perinatal pregnant women infected group B Streptococcus on maternal and infant prognosis. Chin J Nosocomiol, 2016,26(20):4698-4700. |
| 15 | XU Shuiqing, et al. Drug resistance of Streptococcus agalactiae causing urogenital tract infections in pregnant women. Chin J Nosocomiol, 2015,25(16):3788-3789+3807. |
| 16 | WANG Xin, et al. Analysis of infection and drug sensitivity of streptococcus agalactis in pregnant women. The Journal of Practical Medicine, 2013,29(10):1682-1683. |
| 17 | SHI Xingquan, et al. Analysis and clinical significance of drug resistance of Group B Streptococcus from pregnant women with perinatal genitourinary tract infection. Chinese Joumal of Microecology, 2013,25(08):966-968. |
| 18 | TIAN Xuqin, et al. Influence of vaginal Group B Streptococcus infection on prematurer upture of fetal membranes. Chin J Nosocomiol, 2017,27(09):2107-2110. |
| 19 | LIU Jing, et al. Resistance analysis of group B streptococcus agalactiae in pregnant women. Chin J Health Lab Tec, 2013,23(16):3298-3299. |
| 20 | HU Falong, et al. Streptococcus agalactiae infection and drug resistance in women in late pregnancy , and the influence on newborns. Chin J Microecol, 2016,28(05):598-600. |
| 21 | KUANG Linghan, et al. Epidemiology and Resistance Mechanisms of Group B Streptococci in Late-pregnant Maternal Birth Canal. J Sichuan Univ (Med Sci Edi), 2015,46(05):692-696. |
| 22 | YANG Xiaoyan, et al. Result Analysis on Bacterial Culture and Drug Susceptibility of Fetal Membranes in Women with Premature Rupture of Membranes. Journal of Practical Obstetrics and Gynecology, 2018,34(03):223-227. |
| 23 | SONG Haiyan, et al. Colonization prevalence, antimicrobial resistance and serotypes of Group B Streptococcus isolates in late pregnancy in Qingdao. Chin J Lab Diagn, 2019,23(11):1937-1939. |
| 24 | LIU Hongying, et al.. Infection of Group B Streptococcus in Vaginal Tract of Perinatal Pregnant Women and Its Influence on Pregnancy Outcomes. Labeled Immunoassays & Clin Med,2018,25(05):707-709. |
| 25 | HOU Yaping, et al.Analysis effect of group B Streptococcus infection and drug resistance in third trimester gestation women at Changning District of Shanghai. Lab Med Clin,2018,15(14):2132-2133+2136. |
| 26 | SU Jinzhen, et al.Drug resistance , serotypes , virulence-associated genes and genotypes of infection or colonization of group Bstreptococcus in perinatal pregnant women. Chin J Obstet Gynecol Pediatr ( Electron Ed ),2016,12(05):583-589. |
| 27 | WU Airong, et al.Effect Evaluation and Drug Resistance Analysis of B Streptococcus and Fungus in Swab of Vaginal and Perianal Detected with Different Methods in Late Pregnancy. J Mod Lab Med,2018,33(03):108-111. |
| 28 | XU Lijuan, et al. Investigation on Group B Streptococcus Carried by Women in Late Pregnancy in Lanzhou City and Analysis of Drug Sensitivity.Lab Med Clin,2019,16(15):2188-2189. |
| 29 | JI Tongzhen, et al. Screening of Group B Streptococcus agalactis in pregnant women and comparative analysis of drug sensitivity. Chin J Lab Diagn, 2016,20(12):2072-2073. |
| 30 | XU Ping, et al. Colonization of group B streptococcus in late-pregnant women and its relation with perinatal outcomes. Zhejiang Medicine, 2020,42(08):851-853. |
| 31 | WANG Shuangjie, et al. Analysis of Group B Streptococcus Infection in Pregnant Women and Infants in Late Pregnancy. Guangxi Medical journal, 2017,39(12):1925-1927+1942. |
| 32 | ZHANG Kaier, et al. Group B Streptococcus Infection in the Reproductive Tract of Pregnant Women in Late Pregnancy and Analysis of Pregnancy Outcome. Maternal and Child Health Care of China, 2020,35(01):117-119. |
| 33 | ZHANG Yu, et al. Group B streptococcus infection in late pregnancy and the influence of intervention on pregnancy outcome. Lab Med Clin, 2018,15(13):1974-1977. |
| 34 | QIAN Yaoxian, et al. Carrying situation of group B streptococcus in pregnant women and its effect on newborn outcome. Lab Med Clin, 2019,16(14):1969-1971+1975. |
| 35 | DU Wenyuan, et al. Effect of group B streptococcus infection on pregnancy outcome in latepregnancy and analysis of drug resistance. Chinese Journal of Family Planning & Gynecotokology, 2019,11(03):64-67. |
| 36 | WANG Li, et al. Infection characteristics and drug resistance of group B streptococcus in perinatal pregnant women. Lab Med Clin,2019,16(11):1575-1578. |
| 37 | XIE Wen, et al. Analysis of Group B Streptococcus in Perinatal Pregnant Women in Changsha and Its Effect on Pregnancy Outcome. Journal of Hunan University of Chinese Medicine, 2018,38(05):590-592. |
| 38 | XIE Yun, et al. Changes of Antibiotic Resistance and Serotype Distribution of Group B Streptococcus in the Reproductive Tract of Pregnant Women during Perinatal Period. J Int Obstet Gynecol,2019,46(06):657-660. |
| 39 | YANG Hongya, et al. Detection of Group B Streptococcus Infection and Drug Resistance in Perinatal Pregnant Women and Its Influence on Pregnancy Outcome. Pract Prey Med,2018,25(04):502-504. |
| 40 | ZENG Baihua, et al. Analysis of the colonization and high r isk factors of group B Streptococcusen in the third trimester of pregnancy. J Xuzhou Med Univ, 2017, 037(010):658-660. |
| 41 | WANG Zhenrong, et al. Clinical Analysis of Pregnant Women Infected With Genital Streptococcus Agalactiae. Chinese Journal of Coal Industry Medicine, 2017,20(03):266-268. |
| 42 | ZHANG Luyan, et al. The relationship between the colonization of Streptococcus agalactis and premature rupture of membranes in perinatal women and the analysis of drug resistance. Chinese Clinical Doctor,2018,46(07):858-860. |
| 43 | TANG Xingmin, et al. Analysis of drug resistance of Streptococcus agalactis isolated from the genitourinary tract of pregnant women. Maternal and Child Health Care of China,2017,32(11):2425-2427. |
| 44 | ZHANG Hua, et al. Analysis of Group B Streptococcus Infection and Drug Resistance in Perinatal Pregnant Women. International Journal of Laboratory Medicine,2017,38(11):1543-1545. |
| 45 | GAO Shuang, et al. Investigation of Different Serotype Distribution of Group B Streptococcus in Women with Late Pregnancy in Shenyang. Journal of International Obstetrics and Gynecology,2019,46(05):515-518+522. |
| 46 | ZHANG Qian, et al. Effect of Genital Tract Group B Streptococcus( GBS) Infection in Late Pregnancy on Pregnancy Outcomes. HEBEI MEDICINE,2018,24(04):608-612. |
| 47 | RONG Lili, et al. Genotyping and Molecular Epidemiology Investigation of GBSPathogenic Strains of GBS Positive Pregnant Women in Guangzhou. J Mod Lab Med,2017,32(01):87-90. |
| 48 | CHEN Jingqun, et al. Perinatal maternal group B streptococcal infections and their effects on mother and child. Pract Prey Med,2017,24(03):349-351. |
| 49 | WANG Fengping, et al. Prenatal screening and drug resistance research of group B Streptococcus in perinatal pregnant women. Chin J Health L ab Tec, 2017,27(07):994-996+999. |
| 50 | LIU Jialing, et al. Screening of Streptococcus Agalactiae of Genitourinary Tract in the LatePregnant Women in Guizhou Province and the Genetic Sequencing Analysis. J Mod Lab Mad,2018,33(01):14-18. |
| 51 | WANG Fenfang, et al. Analysis of infection types and drug resistance of group B Streptococcusin perinatal pregnant women and newborns. Chin J Health Lab Tec,2016,26(23):3469-3470+3473. |
| 52 | WU Hongguang, et al. Group B Streptococcus Infection in Vaginal Tractof Perinatal Pregnant Women and Drug Resistance Analysis. J Mod Lab Med, 2016(1):4. |
| 53 | ZHANG Ying, et al. Analysis of epidemiological characteristics and drug resistance surveillanceresults of Group B Streptococcus infection in perinatal pregnant women. Chin J Health Lab Tec,2017,27(16):2369-2372. |
| 54 | ZHENG Jianqiong, et al. Influence of adequate intrapartum antibiotic prophylaxis on neonatal outcomes in pregnant women withpositive group B streptococcus colonization. Chinese Journal of Practical Gynecology and Obstetrics,2018,34(06):675-679. |
| 55 | LIU Jing, et al. Analysis of drug sensitivity of hospitalized pregnant women infected with Streptococcus agalactis. Chin J Lab Diagn, 2015,19(12):2110-2111. |
| 56 | LI Yamei, et al. Detection of Group B Streptococcus and Analysis of Drug Resistance in Perinatal Pregnant Women and the Influence of the Pregnancy Outcome. J Mod Lab Med, 2013(1):3. |
| 57 | MA Danjuan, et al. The distribution of bacteria of reproductive tract infection and analysis of colonization of group B Streptococcus in women during perinatal period. Maternal and Child Health Care of China .,2016,31(03):565-567. |
| 58 | ZHANG Lihua, et al. Detection rate and drug resistance of group B streptococcus in pregnant women during perinatal period. China Medicine, 2010, 5(3):261-262. |
| 59 | DUAN Xiaoling, et al.Detection of Streptococcus agalactis in pregnant women in Jinshan District, Shanghai and analysis of drug sensitivity. Chinese joumal of healthy Birth & child care, 2014, 20(008):555-556. |
| 60 | HUANG Lairong, et al. Detection of streptococcus agalactiae and analysis of drug resistance in perinatal pregant women. Lab Med Clin, 2019, 16(S02):5. |
